# Resurrection of a diatom after 7000 years from anoxic Baltic Sea sediment

**DOI:** 10.1093/ismejo/wrae252

**Published:** 2025-01-03

**Authors:** Sarah Bolius, Alexandra Schmidt, Jérôme Kaiser, Helge W Arz, Olaf Dellwig, Ulf Karsten, Laura S Epp, Anke Kremp

**Affiliations:** Biological Oceanography, Leibniz Institute for Baltic Sea Research Warnemünde, 18119 Rostock, Germany; Department of Biology, University of Konstanz, 78464 Konstanz, Germany; International Max Planck Research School Quantitative Behaviour Ecology & Evolution, 78457 Konstanz, Germany; Marine Geology, Leibniz Institute for Baltic Sea Research Warnemünde, 18119 Rostock, Germany; Marine Geology, Leibniz Institute for Baltic Sea Research Warnemünde, 18119 Rostock, Germany; Marine Geology, Leibniz Institute for Baltic Sea Research Warnemünde, 18119 Rostock, Germany; Institute of Biological Science, University of Rostock, 18051 Rostock, Germany; Department of Biology, University of Konstanz, 78464 Konstanz, Germany; Biological Oceanography, Leibniz Institute for Baltic Sea Research Warnemünde, 18119 Rostock, Germany

**Keywords:** phytoplankton, dormancy, resurrection, traits, *Skeletonema*

## Abstract

Dormancy is a widespread key life history trait observed across the tree of life. Many plankton species form dormant cell stages that accumulate in aquatic sediments and, under anoxic conditions, form chronological records of past species and population dynamics under changing environmental conditions. Here we report on the germination of a microscopic alga, the abundant marine diatom *Skeletonema marinoi* Sarno et Zigone, that had remained dormant for up to 6871 ± 140 years in anoxic sediments of the Baltic Sea and resumed growth when exposed to oxygen and light. Resurrected diatom strains, representing cohorts from six different time points of the past 6871 ± 140 years, are genetically differentiated, and fundamental physiological functions such as growth and photosynthesis have remained stable through time despite distinct environmental dynamics. Showing that resurrection and full functional recovery, in comparison to 3 ± 2 years of dormancy, is possible after millennial resting, we emphasize the relevance of dormancy and living sediment archives. For the future, sediment archives, together with the resurrection approach, would offer a powerful tool to trace adaptive traits over millennia under distinct climatic conditions and elucidate the underlying mechanisms.

## Introduction

Across the tree of life, from bacteria to mammals, dormancy is a widespread key trait of life [1]. This allows organisms to survive periods of unfavorable and uncertain environmental conditions by shifting into a reversible state of reduced metabolic activity. Nonetheless, besides securing survival, dormant cells, which are often specialized, have a number of other important features and ecological functions: they contain specialized protective structures such as robust and impregnated cell walls or covers and accumulate storage products to support reduced metabolic activity over long periods of time, thereby promoting survival under unfavorable conditions [2, 3].

Despite their common protective features, dormant cells are highly diverse in their morphologies, with varying shapes and sizes. Different phylogenetic groups build specific forms, like spores, cysts, akinetes, specific eggs, or seeds. These dormant cells or tissues typically differ in morphology from active, vegetative cells [3]. In many organisms, dormant cells are part of the sexual life cycle and facilitate recombination. Additionally, resistant dormant cells can be transported in ecosystems by various vectors, such as migratory birds, animals, and both winds and resuspension by ocean currents, thereby expanding their geographic dispersal and distribution range [4–6].

Sedimentation is a key process that enables dormant cells to form seed or propagule banks, serving as reservoirs of diversity over time and space [1, 7]. When dormant cells accumulate consecutively in undisturbed laminated sediments, e.g. under anoxic conditions, they represent distinct temporal cohorts of respective organisms. Such accumulations constitute so-called “sediment archives” that provide valuable information on past ecosystems, species, and population history, as well as on their response to environmental changes. “Resurrection” ecology [8] refers to the specific germination of dormant cells from such sediment archives, and subsequent characterization of these organisms has proven to be an effective approach to e.g. experimentally study functional traits and their adaptation in relation to environmental changes over time.

The longest dormancy period reported for resting stages buried in sediments so far, lasting for nearly 2000 years, was found for seeds of the date palm *Phoenix dactylifera* from an archeological site [9–11]. Further reports of long-term dormancy of plants range from centuries, such as sedges [12, 13], to 1300 years shown in Sacred Lotus [14]. For aquatic habitats, the oldest record of a resurrected and subsequently growing species stems from the crustacean *Daphnia pulicaria* from a 700-year-old sediment layer of South Center Lake in Minnesota [15]. For phytoplankton, a number of studies reported germination after decadal to centennial periods of dormancy [16–20]. Viable cells germinated from initially dormant cells of diatoms (spores) [21] and cyanobacteria (akinetes) [22] were reported from 6600-years old anoxic Baltic Sea (Landsort Deep) and nearly 2000-years-old French lake sediments, respectively. However, after being released from dormancy, in both studies, the dormant cells remained non-growing due to unknown reasons. But it is reasonable to assume a trade-off between the longevity and viability of the spores/akinetes and their reproductive ability.

Due to recurrent hypoxic (<2 ml L^−1^ O_2_ [23]) to sulfidic (euxinic) conditions in the bottom waters of its deep basins, the Baltic Sea is a particularly suitable system for the application of the sediment archive approach. These conditions, along with cold temperatures, prevent degradation and preserve dormant cells [3]. Several studies on dormancy, resurrection, and survival of phytoplankton dormant cells from Baltic Sea sediments emphasize its specific potential for phytoplankton resurrection after extended time periods [16, 18, 24, 25]. So far, resurrection studies on phytoplankton mostly addressed the period of environmental deterioration of the past ~100 years [16, 18, 20, 24, 25]. However, phytoplankton residues like microfossils (e.g. diatom valves, resting spores) and smaller parts that still contain DNA are constantly found in even older sediment layers [26–28], suggesting that viable phytoplankton cells may also be present for far longer periods of time.

**Figure 1:**
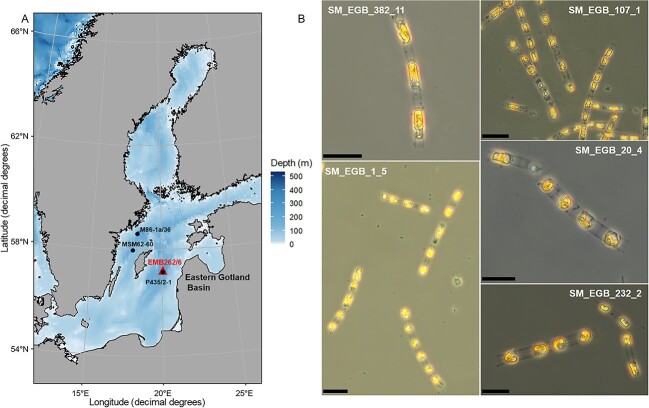
Location of sediment sampling and pictures of resurrected species *S. marinoi*. (A) Map of the Baltic Sea (northern Europe) with the location of sediment core sampling (EMB262/6; 240 m water depth) in the eastern Gotland Basin (triangle). Additional sampling points of stratigraphy reference cores (points): M86-1a/36 [35], MSM62–60 [36], and P435-2-1 [34]. (B) Pictures of different Lugol-stained strains of *S. marinoi*. Strain SM_EGB_382_11 from 6871 ± 140 years of dormancy, SM_EGB_1_5 from 3 ± 2 years of dormancy, SM_EGB_107_1 from 1131 ± 110 years of dormancy, SM_EGB_20_4 from 58 ± 5 years of dormancy, and SM_EGB_232_2 from 3411 ± 120 years of dormancy. Scale bars = 20 μm.

Here, we used an anoxic sediment archive from a deep (240 m water depth) basin of the Baltic Sea (Eastern Gotland Basin, Fig. 1A) represented by a composite sediment core covering the past 7500 years to investigate how far back in time dormant phytoplankton may remain viable in a dormant state and keeps its germination capability. The sediment core represents the mid-to-late Holocene history of the Baltic Sea, the so-called Littorina Sea stage [26], offering the possibility to search for viable phytoplankton dormant cells millennia back in time and specifically using layers that represent the different climate phases and respective conditions of the Holocene. To evaluate the viability and functional recovery of resurrected strains, functional physiological traits such as growth rate and oxygen production were examined. We report the resurrection of a common Baltic Sea spring bloom diatom, *Skeletonema marinoi* Sarno et Zigone (Fig. 1B)*,* from millennial dormancy and demonstrate the millennial viability of dormant strains.

## Materials and methods

### Sediment sampling

During a cruise on RV Elisabeth Mann Borgese (EMB262), in April 2021, a short (54 cm, EMB2625/6–28) and long sediment core (547 cm, EMB262/6–30) were retrieved by a multicorer device and a gravity corer, respectively, from the deep Eastern Gotland Basin (EGB; 57°17.004’ N, 020°07.244′ E, 240 m water depth), Baltic Sea. To prevent the use of disturbed sediments from the uppermost part of the gravity core (~ top 40 cm), both cores were combined into a composite profile. The sediments were nearly entirely laminated, and no signals of bioturbation could be visually identified (Supplemental Material Fig. S1). This finding agrees with the contents of redox-sensitive trace metals determined in sediments from a previous core obtained at an identical location in December 2018 (EMB201). Thus, the corresponding records of manganese and molybdenum indicate alternating hypoxic and euxinic bottom water conditions throughout the sampled brackish Littorina phase (Supplemental Material Fig. S2). Overall, this confirms the undisturbed state of the recovered sediment material used for this study.

To avoid sediment core alterations and contamination, the cores were immediately split and sub-sampled on board, according to sampling procedures for sedimentary ancient DNA [29]. Therefore, the surface of the split core half was carefully cleaned, and the outer sediment that had been in contact with the core liner (~0–0.5 cm) was discarded. For the resurrection of phytoplankton, ca. 3 cm^3^ of the sediment was sampled from one half of the core, using a sterile syringe, at a resolution of 1–2 cm on the short core and every 10 cm on the long core and stored dark at 4–6°C.

### Stratigraphy

The dating of the short core is based on an event stratigraphic approach using, e.g. activities of radiogenic ^137^Cs and ^241^Am from former nuclear bomb testing (onset 1954 and max. 1963), and the Chernobyl accident in 1986, Hg contents for the onset of industrial and modern pollution around 1900 and 1950, stable ^206/207^Pb for maximum pollution from leaded fuel (1970–80), respectively, and Mn contents indicating Major Baltic Inflows (MBIs) in 1994, 2003, and 2014 [30–33]. The present short core was taken from the same location where two independently dated short cores from a previous cruise (EMB201, December 2018) were available [32, 33], allowing age model transfer via core parallelization. Sediment core regions that are disturbed were not used for resurrection approach, as, for example, seen in cm 162–172 of EMB262/6–30 (GC; Supplemental Material Fig. S2).

**Figure 2:**
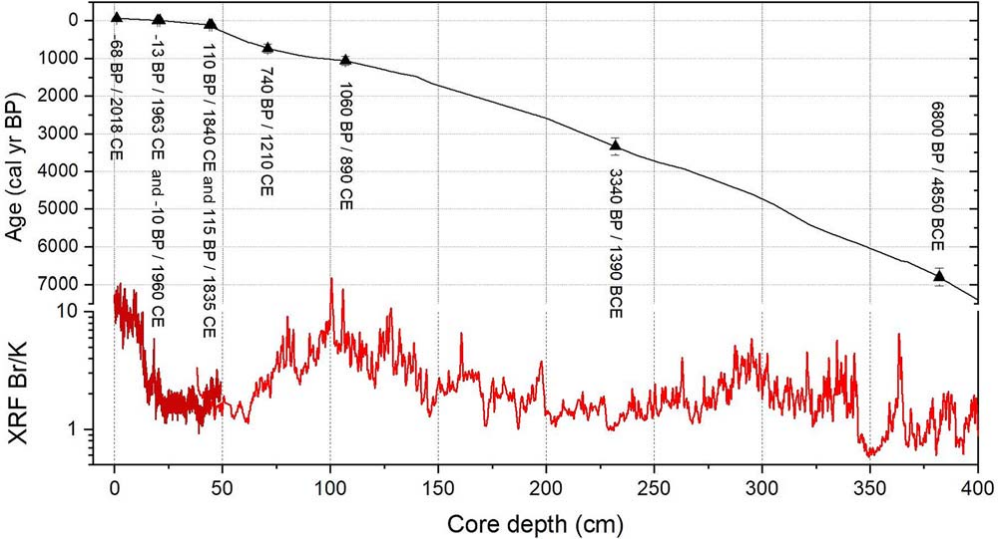
Age-depth model of core EGB262/6 from the eastern Gotland Basin with the age of resurrected temporal cohorts of *S. marinoi* (triangles). The Br/K ratio of the sediments reflects the relative organic matter content of the sediment [60], with high (low) Br/K values indicating high (low) contents. Calculated years ± root mean squared errors. Years in (calculated years) BP (before present = 1950) and B/CE (before the/common era) respectively.

The age assignment of sediment core EMB262/6–30-GC (EGB long core; Supplement Material Fig. S3) is achieved through a detailed visual correlation of high-resolution XRF scanner elemental Br/K values. These ratios are compared with loss-on-ignition (LOI) and total organic carbon (TOC) from recently published central Baltic Sea sediment cores owning exceptionally well-dated radiocarbon chronologies. For the older, mid-Holocene part, the chronology of sediment core P435–2-1 core at about the same location as EMB262/6–30 in the EGB (Fig. 1A) was used as a reference [34]. For the late Holocene part (last ~3000 years), sediment cores M86-1a/36 [35] (Fig. 1A) and MSM62–60 [36] (Fig. 1A) from the western Gotland Basin served as reference chronologies. A precise age assignment of pre-Littorina-Stage sediments was not possible, but the lowermost, about 50 cm of the core most likely belonged to the late Ancylus Lake phase <9500 calibrated years Before Present (cal yr BP, present = 1950 CE) of the Baltic Sea. The short and long cores were finally combined into one composite core (EMB262/6; text above, Fig. 2). Root mean squared errors were calculated to estimate chronological uncertainties. Uncertainties of the reference chronologies, sample spacing of the reference and own records, and an extra uncertainty allowance for more ambiguous tie-points were considered [37].

### Resurrection and cultivation of *Skeletonema marinoi*

For the resurrection approach, sediments from 12 sampled and well-defined layers of the core were selected to represent the main climate phases of the Baltic Sea Holocene history. Samples were taken from cm 1 (−68 ± 2 cal yr BP), 3 (−63 ± 1 cal yr BP), 16 (−24 ± 4 cal yr BP), 20 (−13 ± 5 cal yr BP), 21 (−10 ± 5 cal yr BP), 35 (66 cal yr BP), 44 (110 ± 15 cal yr BP), 45 (115 ± 15 cal yr BP), 71 (740 ± 110 cal yr BP), 107 (1060 ± 110 cal yr BP), 232 (3340 ± 120 cal yr BP), and 382 (6800 ± 140 cal yr BP) and thus cover the Holocene Thermal Maximum (HTM; 8000–4000 cal yr BP) with a warmer climate; the Medieval Climate Anomaly (MCA; 1150–600 cal yr BP), warmer climate, but not as warm as HTM; the Little Ice Age (LIA; ~400–100 cal yr BP), a period of cold temperatures, and the ongoing Modern Warm Period (MWP, ~since 100 cal yr BP), with a fast increase in temperature until today [38] (Supplemental Material Table S1).

Around 0.5–1 cm^3^ of each sediment sample was suspended in 15 ml of sterile filtered and autoclaved Baltic Sea water (salinity of 7.5, taken from the Bornholm Basin) and sonicated (45 s at 80%). The resulting sediment slurry samples were diluted 1:100 with f/8 medium with silicate [39] (diluted from f/2, f/2 silicate concentration: 106 μM; using sterile 0.2 μm filtered Baltic Sea water) and distributed in 24-well plates, with 2 ml in each well. For each sediment sample, 4 24-well plates (96 replicates) were prepared and incubated at 4°C, 40 μmol photons m^−2^ s^−1^ photon fluence rate, and a 16:8 h light:dark cycle.

After 6 weeks–3 months, the slurries were diluted 6-fold again with f/8 medium to provide the dormant cells with an additional “nutrient push”. The wells were checked for germinated cells every week under an inverted microscope (100–400 x magnification; Zeiss, Jena, Germany); after ~1–16 months, individual cells of *S. marinoi* were isolated and established from the wells using a thin glass capillary connected to a tube and fully established as cultures after 2–4 re-isolations. The well plates were sealed with Parafilm to avoid any contamination through the air. Furthermore, cross-contamination was excluded by DNA analyses in the course of the study (see Methods, Results). The re-isolations were performed to obtain clonal cultures and minimize bacterial contamination as much as possible. Subsequently, the strains were cultured in f/2 medium with silicate (106 μM) at 4°C, with a 16:8 h light:dark cycle and 40 μmol photons m^−2^ s^−1^ photon fluence rate.

### DNA extraction and microsatellite analysis

To extract DNA, cultures of *S. marinoi* were centrifuged during their (mid) exponential growth phase, and the cell pellets were frozen at −80°C until further processing. For extraction, the Qiagen DNeasy Plant Pro Kit (Hilden, Germany) was used, following the manufacturer’s protocol.

To lyse the cells, the frozen cell pellets were thawed, mixed with the lysis buffer, and subjected to an initial bead-beating for 30 s. Subsequently, 2 μL of proteinase K (Thermo Fisher Scientific, Waltham, MA, USA) was added, and the cells were homogenized in two rounds of 45 s with a 5-min break in between on a FastPrep machine. The lysate was then incubated with rotation at 56°C over-night. The remaining extraction followed the manufacturers’ suggestions, and the DNA was stored at −20°C.

Genetic diversity, based on 8 microsatellites, was analyzed on a subset of 62 DNA extracts (Supplemental Material Table S1). These were diluted to a concentration of 20 ng μL^−1^. The eight primer pairs (S.mar1-S.mar8 [40], Supplemental Material Table S2) were used in a 33 μM concentration. The forward primers were modified with fluorescent dyes at the 5′ end. Modified forward primers were dissolved in a 10 mM Tris–HCl buffer (pH 7.5), and the reverse primers were in diethyl pyrocarbonate (DEPC)-treated water.

The eight primer pairs were combined in three different primer mix combinations: (i) S.mar1–4, (ii) S.mar5–7, and (iii) S.mar8. This resulted in a total of three polymerase chain reactions (PCRs) per sample. Each reaction mix of 25 μL was transferred into a 96-well PCR plate and consisted of five ingredients: (i) 9.5 μL DEPC H_2_O, (ii) 12.5 μL AmpliTaq Gold 360 Master Mix, (iii) 1 μL of forward primer mix, (iv) 1 μL of reverse primer mix, and (v) 1 μL of diluted DNA extract (20 ng μL^−1^).

The PCR plate was transferred into a Primus 96 advanced PCR-Cycler (Peqlab, VWR, Radnor, PA, USA). The following cycling parameters were used: an initial denaturation for 10 min at 95°C, followed by 40 cycles of 95°C for 30 s, 65°C for 30 s, 72°C for 30 s, and a final elongation step at 72°C for 7 min. Afterward, the samples were stored at –20°C.

After performing all PCRs, the labeled products were sent to Microsynth SEQLAB (Göttingen, Germany) for fragment length analysis.

### Ecological traits

As proxies for a successful resurrection, proof of viability, and “functionality” of *S. marinoi*, the growth rate and photosynthetic activity of the isolated strains from different lengths of dormancy were measured. We thereby consider the most recent strains from the youngest sediment layer (in this case −68 ± 2 cal yr BP or 3 ± 2 years of dormancy, from the year of sediment coring 2021) as modern and reference, respectively, according to other resurrection studies [18, 41]. These strains have only bloomed recently and under the currently prevailing environmental conditions.

From isolated clonal cultures of *S. marinoi* (stock cultures—see above) strains from 6 sediment ages (see Supplemental Material Table S1), inoculum cultures were set up during their exponential growth by adding 2 ml culture suspension into 40 ml f/2 medium with silicate in autoclaved Baltic Sea water (salinity 7.5). When these cultures reached their exponential phase, the experiment was started. The starting cell concentration was set up to a low concentration of 3500 cells ml^−1^ (± 427) based on triplicate fluorescence measurement (Plate Reader, infinite m plex Tecan, ext 450 nm, em 682 nm; ~3500 cells ml^−1^  **≙** nine relative fluorescence units (rfu)) and dilution of the inoculum cultures. For each strain, four replicates to follow growth were set up in 24-well plates (2 ml culture). The growth was recorded daily by chlorophyll-*a* (chl-*a*) fluorescence measurement until ca. 3 days after growth ceased (the stationary phase). On average, the strains reached their mid-exponential phase after 10 days and the stationary phase after 15 days. As the mid-exponential phase, the middle in between the start and end of the exponential growth (in days) was taken. The growth rate was measured as average exponential growth. The experiment ran at 4°C with a 16:8 h light:dark cycle and 40 μmol photons m^−2^ s^−1^ photon fluence rate. These were the same conditions as during the germination process. During the growth experiment, the well plates were closed with breathable plate seals (Breath-Easy, Sigma Aldrich, St Louis, MO, USA) to prevent any evaporation.

For the measurement of photosynthetic activity, three strains from four sediment layers were used (Supplemental Material Table S1) to examine photosynthetic activity under saturating light. Again, from stock cultures, inoculum cultures were set up during their exponential growth by adding 2 ml in 40 ml f/2 medium with silicate in autoclaved Baltic Sea water. To measure the photosynthetic activity of the cultures, a so-called “Photosynthetic Irradiance Box” (PI-Box) was used, following a widely applied methodology [42, 43] and mimicking natural conditions. Under a light gradient from 0 (dark) to 1446 ± 83 μmol photons m^−2^ s^−1^, the oxygen production was measured using an optode as a sensor, with 4–8 technical replicates per culture. The light was changed every 10 min without any dark phases, whereby the first and last minutes were not taken into account in the subsequent calculation. At the end of each measurement, particulate chl-*a* determination was undertaken on GF/6 filters (Whatman, 25 mm) to relate oxygen data to chl-*a* cell content. The chl-*a* was extracted in 4–10 ml 70°C hot ethanol for 10 min, centrifuged for 10 min at 4840 × g (Heraeus Megafuge 1.0R, Hanau, Germany), and spectrophotometric measured (extinction of 665 and 750 nm; Shimadzu UV-2401 PC, Kyoto, Japan).

### Calculations and statistical methods

The raw microsatellite data in the form of Fasta-files was analyzed with the R package Fragman [44]. Resulting SSR call results were stored in an R data frame and then further analyzed using the packages tidyverse [45], PolySat version 1.6 [46], and vegan version 2.6–4 [47]. An ANOSIM and PERMANOVA were performed to check if the SSR calls of the cultures differ from each other. For each time point, we calculated observed and expected heterozygosity, the Shannon index, and allele richness across loci. We also evaluated allele size variation across each time point.

The growth rate *μ* was calculated from the start of the exponential growth to the start of the stationary phase, according to:


(1) \begin{equation*} \mu =\frac{\ln \big({RFU}_{ty}\big)-\ln \big({RFU}_{tx}\big)}{tx- ty} \end{equation*}

where RFU_ty_ is the fluorescence of the culture at the end of the exponential phase, RFU_tx_ is the fluorescence of the culture at the beginning of the exponential phase; ty is the day at which the culture is at the end of the exponential phase, tx the day when the culture is at the beginning of the exponential phase.

**Figure 3:**
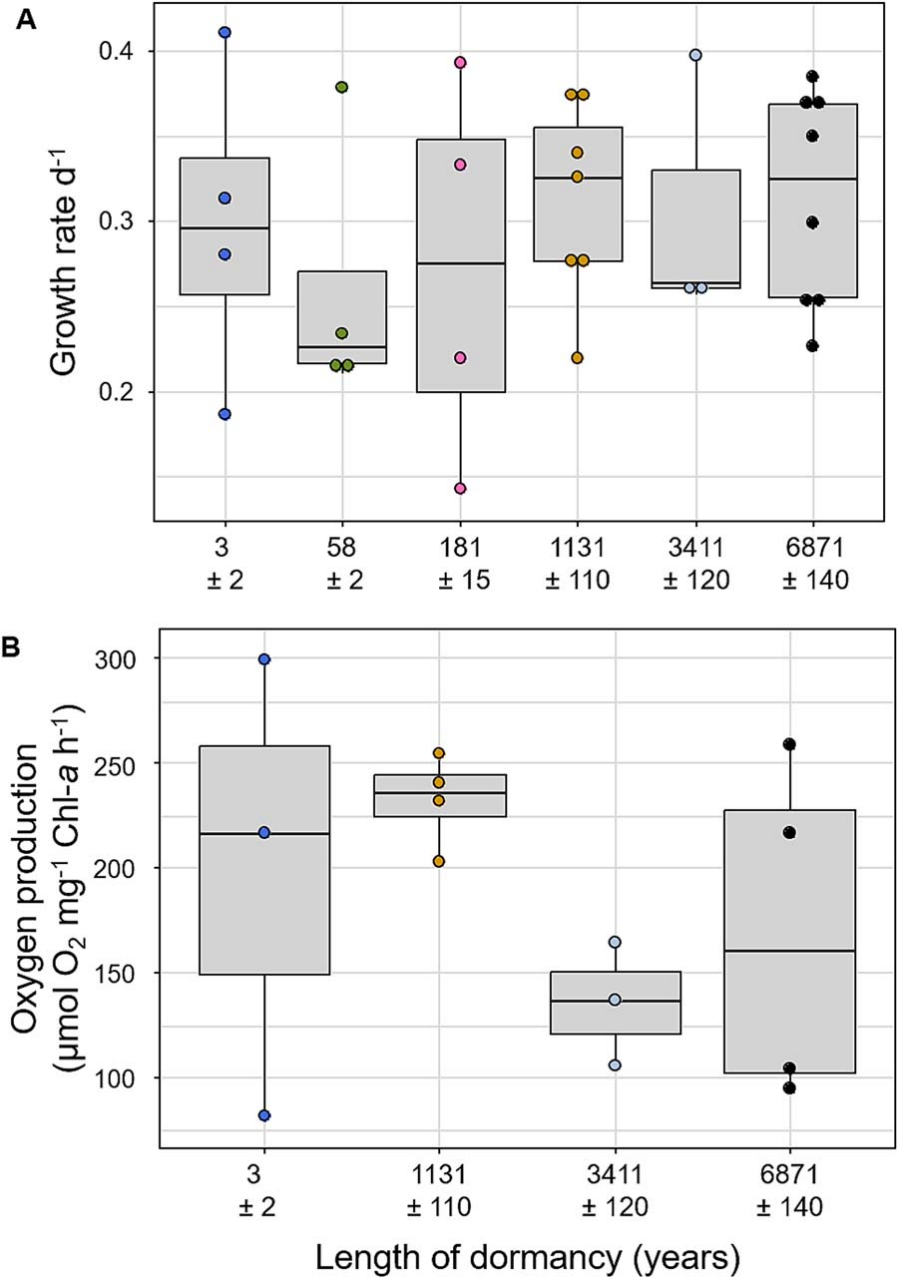
(A) Mean growth rate of *S. marinoi* temporal cohorts from 3 ± 2 (*n* = 4), 58 ± 5 (*n* = 4), 181 ± 15 (*n* = 4), 1131 ± 110 (*n* = 7), 3411 ± 120 (*n* = 3), and 6871 ± 140 (*n* = 8) years of dormancy at 4°C. (B) Mean oxygen production of *S. marinoi* temporal cohorts from -3 ± 2 (*n* = 3), 1131 ± 110 (*n* = 4), 3411 ± 120 (*n* = 3), and 6871 ± 140 (*n* = 4) years of dormancy at 4°C.

To obtain oxygen production data (Fig. 3B and Supplemental Material Table S3), these raw data were fitted with a widely used model for photosynthesis by estimating from least-square regression curves to data [48], using the solver function in MS Office Excel 2016.

To test growth and oxygen production data for normal distribution and subsequent statistical analysis (analysis of variance (ANOVA) or Kruskal–Wallis), R was used.

## Results

The core (EMB262/6) obtained in EGB for the resurrection approach covers the history of the Baltic Sea from a modern, recent time point (here −68 ± 2 cal yr BP/2018 CE/3 ± 2 years of dormancy) to ~7500 years ago (Fig. 2).

Of the 12 sediment layers analyzed for resurrection capacity (see Methods), we were able to resurrect actively growing strains of the chain-forming diatom *S. marinoi* from nine sediment layers (~70%; Supplemental Material Table S1). Resurrection success was relatively low, though, as growing isolates could only be successfully established from ca. 10% of incubated sediment slurries. Nevertheless, the established strains from distinct sediment layers represent a time frame of nearly 7000 years, ranging from 3 ± 2 years (−68 ± 2 cal yr BP) to 6870 ± 140 years of dormancy (Fig. 2). Here, we consider the most recent strains (3 ± 2 years of dormancy) as modern strains, as they bloomed during modern times, and use that as a comparison. Temporal cohorts of *S. marinoi* were successfully resurrected from sediment layers dated to −68 ± 2, −13 ± 5, −10 ± 5, 110 ± 15, 115 ± 15, 740 ± 110, 1060 ± 110, 3340 ± 120, and 6800 ± 140 cal yr BP (Fig. 2; Supplemental Material Table S1), which can be translated into dormancy length (from year of sediment coring 2021) of 3 ± 2, 58 ± 5, 61 ± 5, 181 ± 15, 186 ± 15, 811 ± 110, 1131 ± 110, 3411 ± 120, and 6871 ± 140 years. *S. marinoi* was the only phytoplankton species consistently found in the investigated sediment layers, whereas other species appeared mostly in the top layers (Supplemental Material Table S4).

To first ascertain that the successfully isolated strains were distinct, i.e. no repeated strains were established, and the results were not confounded by cross-contamination among the depths [49], we subjected the established strains to microsatellite analyses. Analysis of the simple sequence repeats (SRR) markers (Supplemental Material Fig. S4) revealed that all strains were individually differentiated strains and formed temporal cohorts, providing evidence that we are working with individual strains. The PERMANOVA analysis yielded an *R*^2^ value of 0.28 (*P* = .001) when age was considered as a variable, indicating a significant difference among ages. Similarly, the ANOSIM test resulted in an *R*^2^ value of 0.25 (*P* = .001), further confirming the observed differences as significant. These results provide evidence that the cohorts from different sediment layers are genetically different. Genetic diversity indices, including heterozygosity, Shannon index, and allele richness, demonstrated consistent patterns over time, with minimal variation. Allele size exhibited slight shifts in position across the different lengths of dormancy (time points), but stable patterns within each age group indicate limited allele size variability within each age layer. We are thus confident that cross-contamination among the samples during sampling and laboratory work did not affect our results.

As proof of full functional recovery of the established strains, the growth rate and oxygen production of the oldest established temporal cohorts were compared to the more recent cohort(s) under the culturing conditions. Growth is an important physiological process as it integrates all positive and negative influences on a cell.

For further analyses and characterization of the isolated strains, only a subset of temporal cohorts with >1 strain was considered, i.e. the strains with dormancy of 3 ± 2, 58 ± 5, 61 ± 5, 181 ± 15, 186 ± 15, 811 ± 110, 1131 ± 110, 3411 ± 120, and 6871 ± 140 years (Supplemental Material Table 1).

The growth rate of the temporal cohorts varied on average between 0.26 μ d^−1^ (58 ± 5 years of dormancy) and 0.31 μ d^−1^ (1131 ± 110, 3411 ± 120, 6871 ± 140 years of dormancy; Fig. 3A) at 4°C and did not exhibit any significant variation among temporal cohorts (ANOVA, *P* = .37; Supplemental Material Table S5), suggesting a similar growth potential over dormancy length. Furthermore, in terms of photosynthetic activity, the strain’s length of dormancy of 3 ± 2, 1131 ± 110, 3411 ± 120, and 6871 ± 140 years showed a maximal oxygen production on approximately the same levels from on average between 136 ± 24 μmol O_2_ mg^−1^ chl-*a* h^−1^ (mean and standard deviation; Fig. 3B; 3411 ± 120 cal yr BP) and 224 ± 14 μmol O_2_ mg^−1^ chl-*a* h^−1^ (mean and standard deviation; Fig. 3B; 1131 ± 110 cal yr BP), averaging around 184 ± 66 μmol O_2_ mg^−1^ chl-*a* h^−1^ (mean and standard deviation; Fig. 3B; ANOVA, *P* = .281; Supplemental Material Table S5; further data in Supplemental Material Table S3), with variability among strains.

## Discussion

In this study, we provide evidence for millennial dormancy, viability, and full physiological recovery with uncompromised fitness of a diatom, *S. marinoi,* after 6871 ± 140 years of dormancy in Baltic Sea sediments. This represents, to the best of our knowledge, the longest sedimentary dormancy period reported so far for a eukaryotic organism.

A potential for long-term dormancy, exceeding decadal and even centennial time scales, has been indicated recently for phytoplankton [21]: germination of a 6600-year-old diatom *Chaetocerus muelleri* var*. subsalsum* was shown from permanently anoxic laminated sediments from the Landsort Deep, a comparable basin within the Baltic Sea. However, while metabolically active cells germinated in their study [21], viable growing strains were never established. Nevertheless, their results strongly support our findings, suggesting that long-term survival through resistant dormant propagules may be more common in phytoplankton than expected so far. The resurrected cells of both studies originated from sediment deposited during the HTM, a period of relatively warm climate and high diatom productivity [34, 50]. More (comparative) studies on diatoms and other phytoplankton species with different ecophysiological traits are needed to get deeper insights into the survival potential of phytoplankton in general. In the present investigation, we did not analyze the length of dormancy longer than 6871 ± 140 years; thus, *S. marinoi* and other phytoplankton species could even have potentially longer dormancy periods than shown here or for *C. muelleri* var*. subsalsum* [21].

Although the physiological basis of extended dormancy is still largely unknown, there is evidence for physiological activity in the dormant cells. Respective studies have reported that during dormancy, *S. marinoi* assimilates nutrients through heterotrophic capacities and transforms them into organic matter, even under dark and anoxic conditions [51, 52], which might explain their ability to remain viable over such a long-time span.

Even though germination after long-term dormancy is observed, not much is known about how dormant life under anoxic conditions affects the respective organism after resurrection. All strains tested here grew and performed photosynthesis with only slight differences between contemporary strains and those germinated after multimillennial dormancy. Thus, the present results demonstrate that an extended dormancy period, compared to short dormancy periods of 3 ± 2 years, does not necessarily have detectable impacts on the physiological performance of resurrected cells. The example of *S. marinoi* reported here shows that complex metabolic machinery can apparently re-establish after prolonged periods of significant metabolic reduction and suggests that organisms can resume full functioning after extended dormancy periods, comparable to that of contemporary individuals. This emphasizes the ecological significance of dormancy and its assumingly complex mechanisms. More studies among different species are, again, necessary.

Phases of resting over short periods of time are an appropriate survival strategy in seasonally variable habitats, such as the Baltic Sea. As a consequence of long-term dormancy periods that extend beyond seasonality and under successive sedimentation, dormant cells are cut off from the possibility of resuspension. However, randomly occurring mixing events could potentially lead to the resuspension of ancient dormant cells and could potentially secure survival during catastrophic events, as suggested by Ribeiro *et al.* (2011), demonstrating the enormous resilience potential of phytoplankton. Moreover, sediment archives, dormancy, and subsequent resurrection of species over a millennial time period, as shown here, present a significant opportunity to study adaptation and evolution over far longer than previously possible time periods *in vivo*.

Under the conditions tested here (4°C, salinity 7.5), all revived strains exhibited similar trait value ranges, primarily proving strain viability after extended dormancy and indicating growth capacities comparable to modern individuals despite different conditioning by past warm or cold periods. During the Holocene history, globally [53–55] and in the Baltic Sea (Supplemental Material Table S1, see Methods and [38, 56, 57]), phases of varying environmental conditions, specifically for temperature, salinity, and nutrient availability, have occurred along with increasing human-induced changes [58]. Despite being isolated from sediment layers representing different climatic conditions, significant differences in trait value range among temporal cohorts were not detected for the one tested laboratory condition, suggesting that significant changes in the specific traits examined here were not altered beyond the indicated ranges over 6871 ± 140 years of dormancy. Though somewhat unexpected, this is in line with observations of resurrected phytoplankton over an up to 100-year time span [41, 59] under one condition. While currently difficult to explain, our results might be related to the fact that the experimental conditions, which represent current distribution patterns of *S. marinoi* in the Baltic Sea, are close to their actual minimum temperature [41]. Using the resurrection approach under the conditions defined here most likely represents only a snapshot of the actual niche of the *S. marinoi* population in the Baltic Sea in the intended years. It is likely that selection by laboratory conditions contributed to the outcome in terms of strains and traits being selected originally. As the here used controlled abiotic conditions were also identical during germination and culturing, acclimation to these particular parameters could have taken place. Another factor with implications for selection is seasonality, an aspect that might have shaped temperature adaptations of the respective resurrected cohorts in our study. It is impossible to resolve seasonal bloom patterns of the past that might have driven adaptation to temperature. Over an environmental gradient, trait values for the strains from respective prevailing conditions likely would differ, as recently shown for *S. marinoi* over a period of 60 years [41]. The observed indicated differences in oxygen production are mainly due to variability among the different strains of temporal cohorts. The cohorts with length of dormancy of 1131 ± 110 and 3411 ± 120 years show less variability than strains of the other temporal cohorts.

Taking all our results together and considering the careful handling of all steps from sampling to cell isolation, we are confident of our approach and time frame. The mostly alternating hypoxic/euxinic conditions (Supplemental Material Fig. S2) and the consequently resulting absence of bioturbation, as also indicated by pronounced lamination, emphasize that sediment mixing had not occurred throughout the core (Supplemental Material Figs S1 and S2). Further support comes from the microsatellite analysis, confirming significant delineation of distinct temporal cohorts (Supplemental Material Fig. S4).

Although the data shown here report an extraordinary capacity for life, we are fully aware that substantial research is needed to elucidate the underlying mechanisms of extremely long dormancy, survival, and subsequent germination in phytoplankton and living organisms in general. Nevertheless, the system we describe here for *S. marinoi* can be used to further explore fundamental mechanisms of adaptive evolutionary processes and factors shaping their outcome. First studies offer insights into metabolic processes facilitating centennial dormancy and activation after extended periods of resting [51].

In summary, our study demonstrates the (i) biological significance of dormancy and the enormous potential of phytoplankton to persist over long time scales and survive unfavorable conditions. In addition, it emphasizes (ii) a significant resilience of phytoplankton toward climatic fluctuations. The system established here could provide a powerful tool to trace potential ecological and genetic adaptation patterns on a millennial time scale over periods of altered climatic conditions, offering valuable information on these organisms as a model species and predictions for future climatic changes.

## Supplementary Material

SupplementaryInformation_Bolius_updated_wrae252

## Data Availability

Trait data that support the findings of this study are openly available at https://doi.org/10.5281/zenodo.12723950.
