# Supplementary material for: Resurrection of a diatom after 7000 years from anoxic Baltic Sea sediment
Source: ISME J. 2025 Jan 3;19(1):wrae252. doi: 10.1093/ismejo/wrae252 (PMC11742256; doi:10.1093/ismejo/wrae252)
Supplement: SupplementaryInformation_Bolius_updated_wrae252 [file supplementaryinformation_bolius_updated_wrae252.doc]

**Supplementary Material**

**Resurrection of a diatom after 7000 years from anoxic Baltic Sea sediment**

Sarah Bolius^1*^, Alexandra Schmidt^23^, Jérôme Kaiser^4^, Helge W. Arz^4^, Olaf Dellwig^4^, Ulf Karsten^5^, Laura S. Epp^2^, Anke Kremp^1^

^1^ Biological Oceanography, Leibniz Institute for Baltic Sea Research Warnemünde, 18119 Rostock, Germany

^2^ Department of Biology, University of Konstanz, 78464 Konstanz, Germany

^3^ International Max Planck Research School Quantitative Behaviour, Ecology & Evolution, 78457 Konstanz, Germany

^4^ **Marine Geology,** Leibniz Institute for Baltic Sea Research Warnemünde, 18119 Rostock, Germany

^5^ Institute of Biological Science, University of Rostock, 18051 Rostock, Germany

* Sarah Bolius, Biological Oceanography, Leibniz Institute for Baltic Sea Research Warnemünde, Seestr. 15, 18119 Rostock, Germany
+49 381 5197 3472
Email: sarah.bolius@io-warnemuende.de

| **Core** | **Composite sediment core depth (cm)** | **Year resurrected**  **(cal yr BP)** | **Dormancy period** | **Climate Phase** | **Number of resurrected strains in culture** | **Strains used for analysis** | | |
| --- | --- | --- | --- | --- | --- | --- | --- | --- |
|  |  |  |  |  |  | **Growth rate** | **Photosynthetic data** | **Microsatellite analysis** |
| MUC | 1 | -68 ± 2 | 3 ± 2 | MWP | 4 | SM_EGB_1_3, SM_EGB_1_5, SM_EGB_1_6, SM_EGB_1_7 | SM_EGB_1_3, SM_EGB_1_6, SM_EGB_1_7 | SM_EGB_1_3, SM_EGB_1_5, SM_EGB_1_6, SM_EGB_1_7 |
| MUC | 20 | -13 ± 5 | 60 ± 5 | MWP | 11 | SM_EGB_20_4, SM_EGB_20_5,  SM_EGB_20_8,  SM_EGB_20_11 |  | SM_EGB_20_4; SM_EGB_20_5; SM_EGB_20_6; SM_EGB_20_1; SM_EGB_20_2; SM_EGB_20_3; SM_EGB_20_7; SM_EGB_20_8  SM_EGB_20_9; SM_EGB_20_11 |
| MUC | 21 | -10 ± 5 | 60 ± 5 | MWP | 8 | - | - | SM_EGB_21_1; SM_EGB_21_2, SM_EGB_21_3, SM_EGB_21_4, SM_EGB_21_6, SM_EGB_21_9, SM_EGB_21_12, SM_EGB_21_13 |
| MUC | 44 | 110 ± 15 | 180 ± 15 | LIA |  | SM_EGB_44_3, SM_EGB_44_6, SM_EGB_44_23, SM_EGB_44_24 | - | SM_EGB_44_1, SM_EGB_44_2, SM_EGB_44_3, SM_EGB_44_4, SM_EGB_44_5, SM_EGB_44_6, SM_EGB_44_12, SM_EGB_44_18, SM_EGB_44_19, SM_EGB_44_20, SM_EGB_44_22, SM_EGB_44_23, SM_EGB_44_24 |
| MUC | 45 | 115 ± 15 | 190 ± 15 | LIA |  | - | - | - |
| GC | 71 | 740 ± 110 | 810 ± 110 | MCA | 1 | - | - | SM_EGB_71_1 |
| GC | 232 | 3340 ± 120 | 3410 ± 120 | IP |  | SM_EGB_232_2, SM_EGB_232_3, SM_EGB_232_4 | SM_EGB_232_2, SM_EGB_232_3, SM_EGB_232_4 | SM_EGB_232_2, SM_EGB_232_3, SM_EGB_232_4 |
| GC | 382 | 6800 ± 140 | 6870 ± 140 | HTM |  | SM_EGB_382_2, SM_EGB_382_3, SM_EGB_382_5, SM_EGB_382_7, SM_EGB_382_8, SM_EGB_382_12, SM_EGB_382_11, SM_EGB_382_13 | SM_EGB_382_3, SM_EGB_382_8, SM_EGB_382_11, SM_EGB_382_13 | SM_EGB_382_2, SM_EGB_382_3, SM_EGB_382_5, SM_EGB_382_7, SM_EGB_382_8, SM_EGB_382_9, SM_EGB_382_10, SM_EGB_382_12, SM_EGB_382_11, SM_EGB_382_13 |

**Supplemental Material Table 1**: Depth and associated year/dormancy period in calibrated years before present (cal yr BP, present = 1950 CE; ± error estimate) of resurrected *Skeletonema marinoi* strains from Eastern Gotland Basin. Error estimate of the ages is not including reservoir age uncertainties. Detailed list of strains used for different analsysis. MUC = short core, GC = long core; Climate phases: MWP = Modern Warm Period; LIA = Little Ice Age, MCA = Medieval Climate Anomaly, IP = Intermediate phase; HTM = Holocene Thermal Maximum.


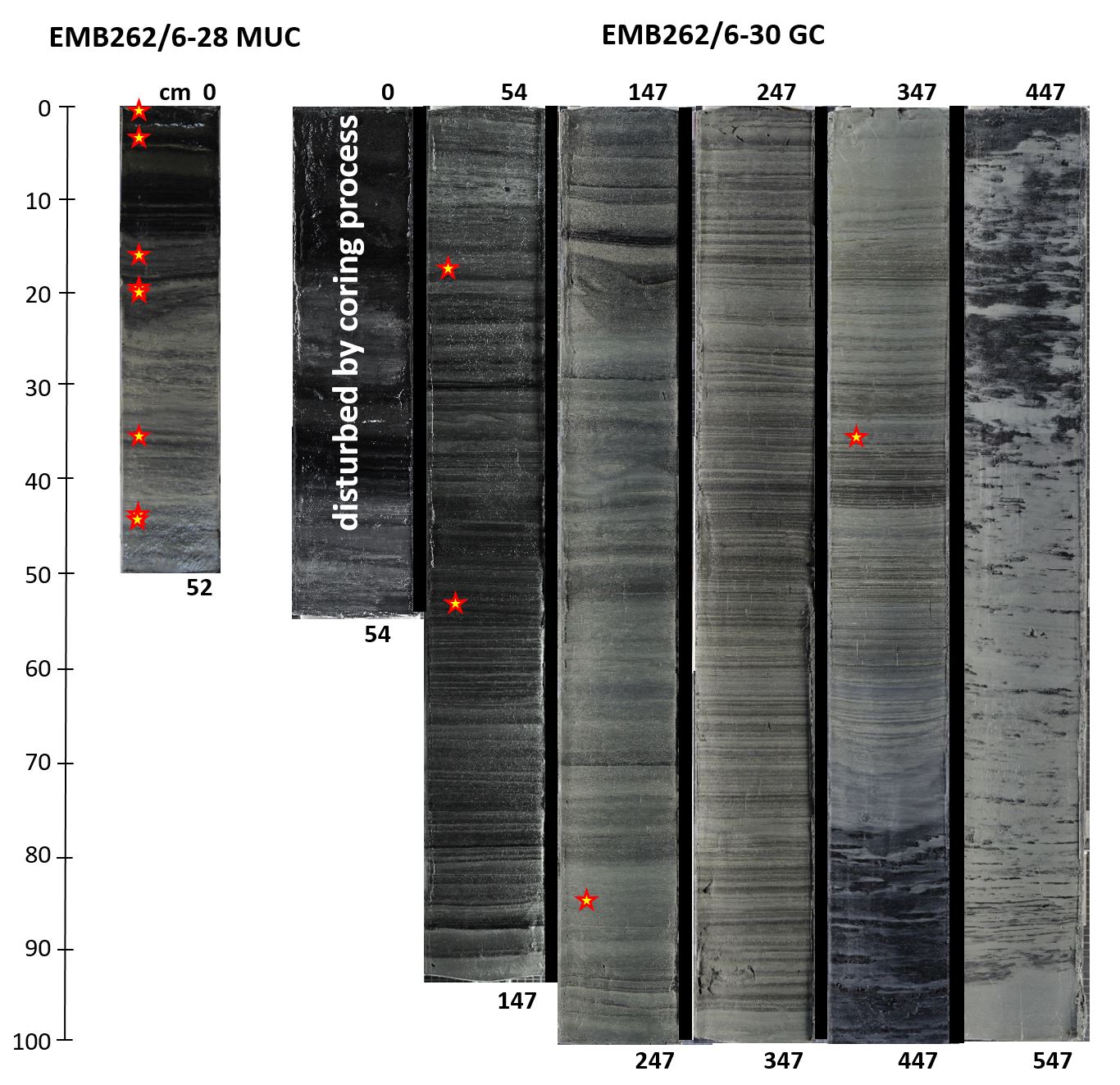


**Supplemental Material Figure 1**: Photos of the sediment cores EMB262/6-8 (MUC) and EMB262/6-30 (GC) from the Eastern Gotland Basin. Sampling depths are marked with red stars and are all located in laminated or at least faintly laminated undisturbed parts of the core.


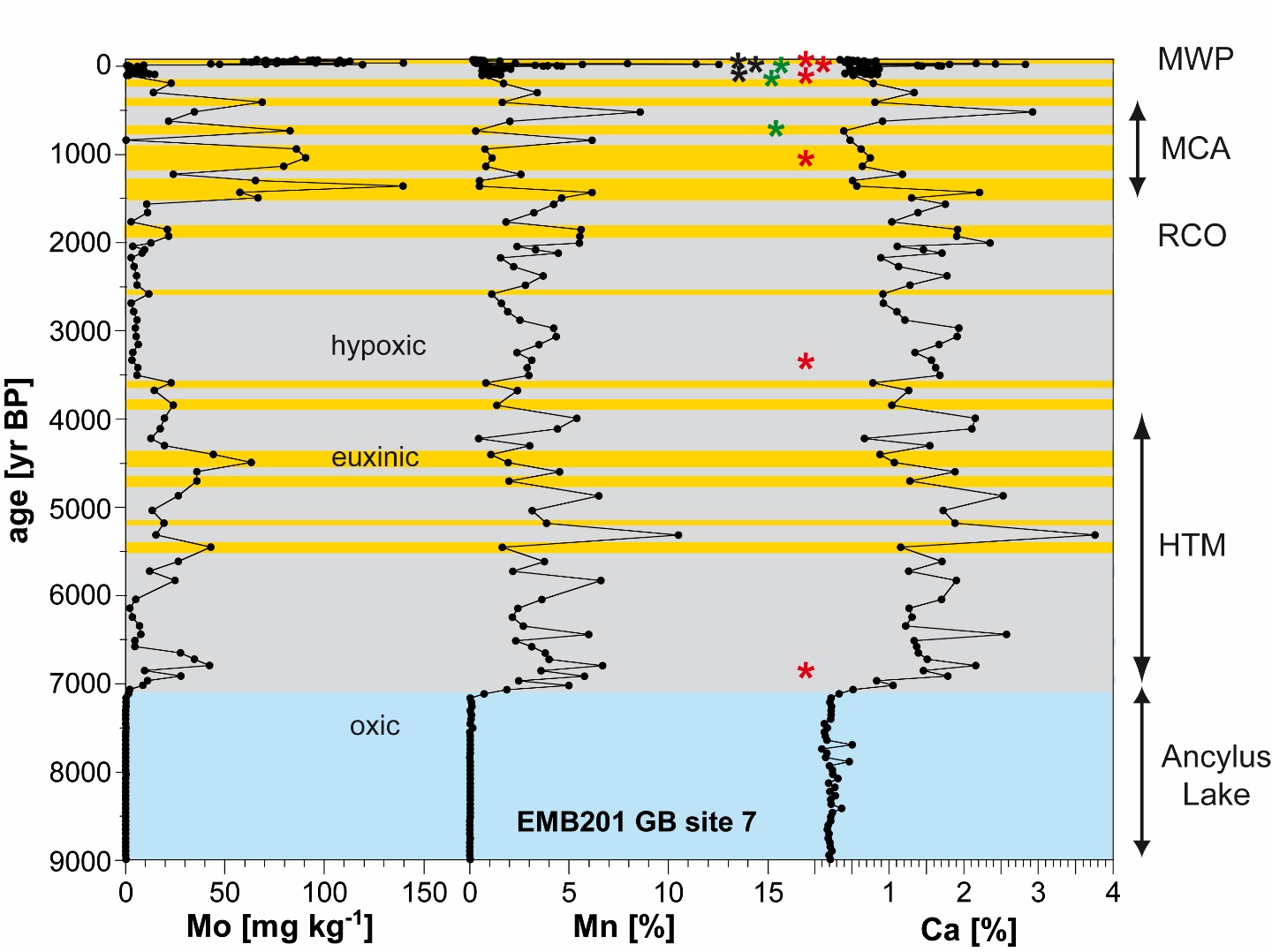


**Supplemental Material Figure 2**: Contents of reactive Mo, Mn and Ca in a composite core (multicorer and gravity core) obtained from the same site in the Eastern Gotland Basin during a former Cruise with RV Elisabeth Mann Borgese (EMB201) in September 2018. Reactive elements were extracted from 200 mg of freeze-dried and homogenized sediment material by shacking with 10 mL 0.1 molar HCl for 1 h at room temperature and were measured for Ca and Mn by ICP-OES and for Mo by ICP-MS. The age model based on the same approach as used for the composite core of this study (MWP = modern warm period, MCO = medieval climate anomaly, RCO = Roman climate optimum, HTM = Holocene Thermal Maximum). Asterisks indicate approx. positions of sediment sampling for diatom investigations (green = resurrected *Skeletonema marinoi*, red = resurrected and growing *S*. *marinoi*). The patterns of the reactive metals suggest that the sampled sediment layers were deposited during periods of alternating hypoxic/euxinic bottom water conditions preventing bioturbation. While strong enrichments of Mo are indicative for euxinia, parallel enrichments of Mn and Ca are due to formation of Ca-rich rhodochrosite, which affords longer-lasting bottom water hypoxia and at least slightly euxinic porewaters (Scott & Lyons, 2012; Dellwig *et al.*, 2018; Häusler *et al.*, 2018).


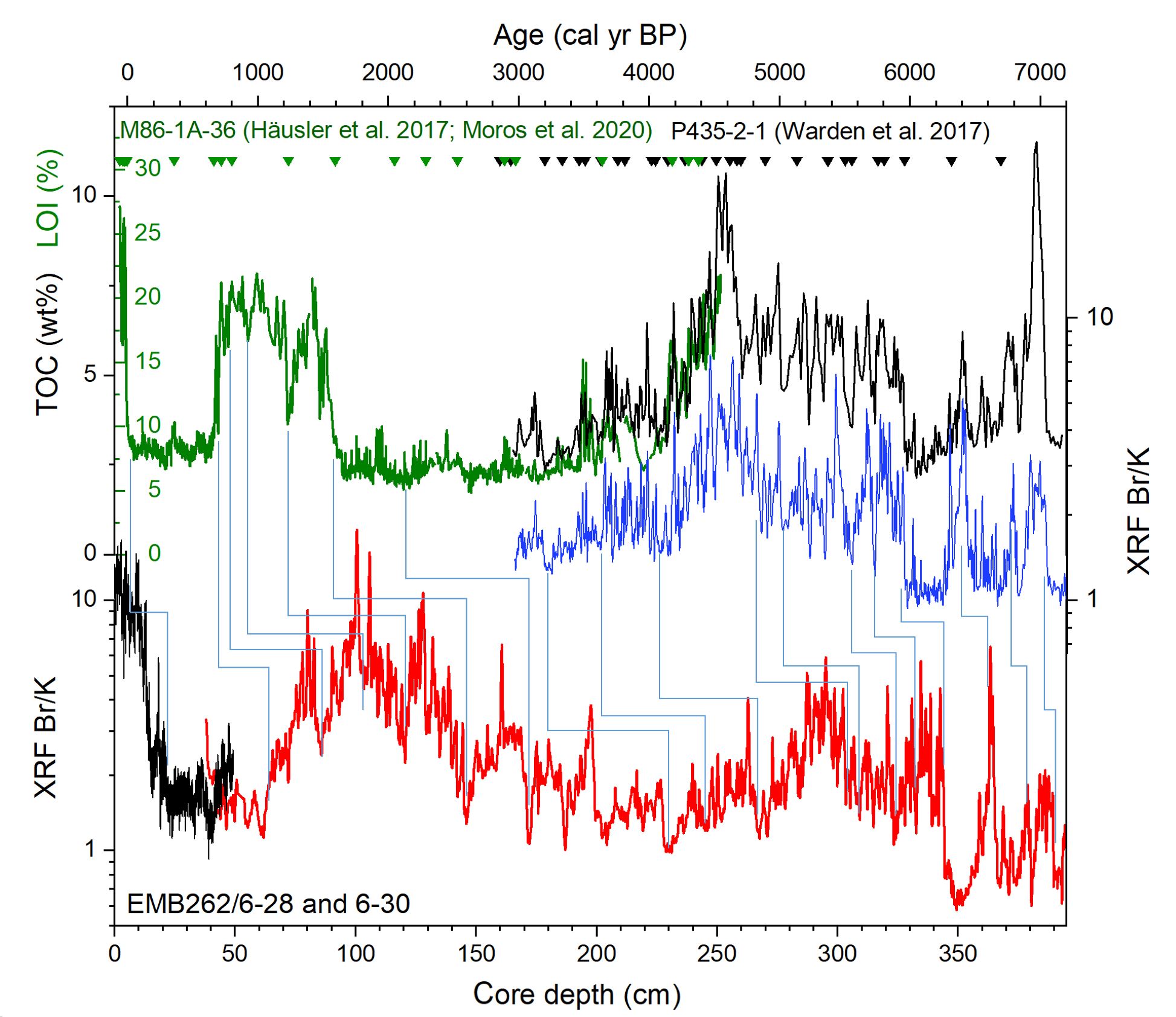


**Supplement Material Figure 3**: Age assignment of sediment cores EMB262/6-28 (MUC, bold black) and EMB262/6-30 (GC, red) through detailed visual correlation of high-resolution XRF scanner elemental Br/K values with loss on ignition (LOI) and total organic carbon (TOC) from published central Baltic Sea sediment cores (Häusler *et al.*, 2018; Moros *et al.*, 2020; green/black/blue). Green and black triangles show age control points of the reference records.


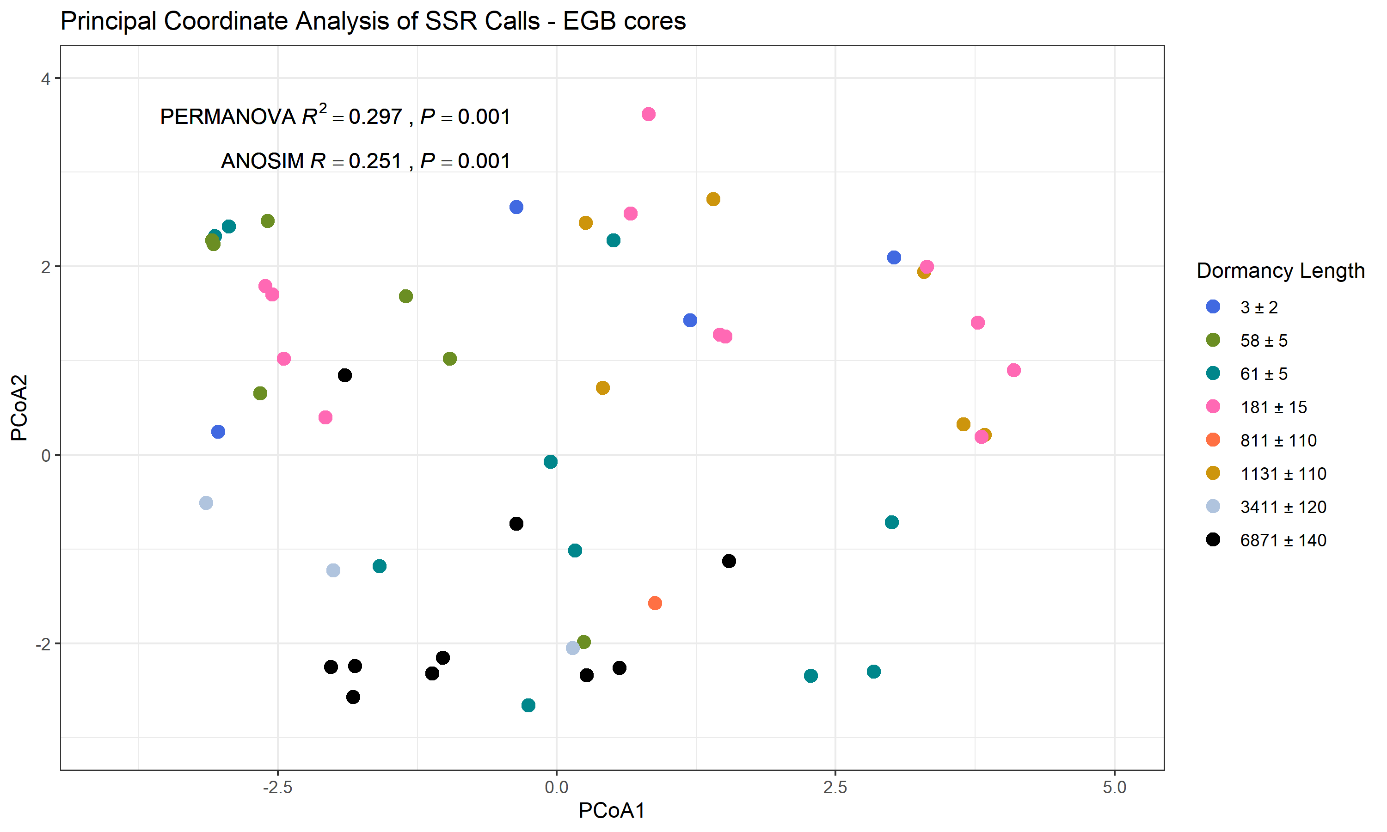


**Supplemental Material Figure 4:** Principal Coordinate Analysis of the simple sequence repeats (SRR) calls for *Skeletonema marioni* re-isolated from the Eastern Gotland Basin (EGB), colours according to the dormancy length of the cohorts.

**Supplemental Material Table 2: Primer and dyes used in the Microsatellite analyses. Primers used from Godhe & Härnström (2010).**

| **Locus** | **Orientation** | **Primer sequence (5'-3')*** |  |  | **Dye** |
| --- | --- | --- | --- | --- | --- |
| S.mar1 | for | GAAAGCAAGTTGAAGTTGAGCGT | | | ATTO565 |
|  | rev | TGTTGATTGAATGAGCGGCTAT | | |  |
|  |  |  |  |  |  |
| S.mar2 | for | TGGGATACGGTCGTTCAA | | | ATTO550 |
|  | rev | CACAACCTTATAATTCAACGCAC | | | |
|  |  |  |  |  |  |
| S.mar3 | for | TTCGGACTTGTTGTATAGGGTGG | | | 6-FAM |
|  | rev | TTGGGTGTGTAGGGTGTTGGT | | |  |
|  |  |  |  |  |  |
| S.mar4 | for | AGGCACTCATGTACTAGATGTCA | | | ATTO532 |
|  | rev | AGGACGAAAGTGAGAGGGT | | |  |
|  |  |  |  |  |  |
| S.mar5 | for | ACCAAAGCATATCGGCACA | | | 6-FAM |
|  | rev | TCAACGCAGCACAGAGAATAGTA | | | |
|  |  |  |  |  |  |
| S.mar6 | for | CTGGTAGCTTTGCTTTGAAGTAA | | | ATTO565 |
|  | rev | ACTCCACCTCCGCATTTT | | |  |
|  |  |  |  |  |  |
| S.mar7 | for | AATGTACCAGTAGAGCTAGAGAGAGAG | | | ATTO550 |
|  | rev | CGTGGTGGATGGTGGATAAT | | |  |
|  |  |  |  |  |  |
| S.mar8 | for | TGCTGCATGAGTGCCTCTAC | | | ATTO532 |
|  | rev | TCCACGAGGAAGAATTGGAG | | |  |

**Supplemental Material Table 3**: Light curve and oxygen production data for all measured strains of *Skeletonema marinoi*.

| **Temporal cohort from year**  (cal yr BP) | **Strain** | **Maximal oxygen production**  P_max_  (µmol O_2_ mg^-1^ Chl-a h^-1^) | **Light saturation point**  l_k_  (µmol photons m^-2^ s^-1^) | **Light compensation point**  l_c_  (µmol photons m^-2^ s^-1^) |
| --- | --- | --- | --- | --- |
| -68 ± 2 | SM_EGB_1_3 | 299.1 | 101.4 | 25.4 |
|  | SM_EGB_1_6 | 217.0 | 79.2 | 33.6 |
|  | SM_EGB_1_7 | 81.7 | 10.3 | 6.9 |
| 1060 ± 110 | SM_EGB_107_1 | 254.7 | 79.1 | 21.1 |
|  | SM_EGB_107_7 | 240.3 | 95.0 | 25.2 |
|  | SM_EGB_107_5 | 232.0 | 111.6 | 15.0 |
|  | SM_EGB_107_4 | 202.9 | 85.7 | 19.5 |
| 3340 ± 120 | SM_EGB_232_2 | 105.6 | 103.9 | 15.9 |
|  | SM_EGB_232_4 | 164.9 | 73.1 | 28.4 |
|  | SM_EGB_232_3 | 137.0 | 38.3 | 20.9 |
| 6800 ± 140 | SM_EGB_382_3 | 104.4 | 56.7 | 29.3 |
|  | SM_EGB_382_8 | 217.0 | 53.0 | 19.0 |
|  | SM_EGB_382_11 | 258.5 | 76.3 | 17.9 |
|  | SM_EGB_382_13 | 94.9 | 67.4 | 24.3 |

**Supplemental Material Table 4**: Further species, in addition to *Skeletonema marinoi* found in the sediment slurries of the Eastern Gotland Basin (Baltic Sea), but not isolated and established as cultures, including benthic species. cal. yr BP = calibrated years before present, BP = 1950.

| **Sediment layer**  **(cal. yr BP)** | **Composite sediment core depth (cm)** | **Phytoplankton species** |
| --- | --- | --- |
| -68 ± 2 | 1 | filamentous cyanobacteria (Dolichospermum sp., *Nodularia* sp.), dinoflagellates (not specified), *Melosira* sp., Scenedesmus *lefevrii*, Thalassiosira spp., centric diatoms (not specified) |
| -13 ± 5 | 20 | *Nitzschia* sp. |
| -10 ± 5 | 21 | *Nitzschia* sp. , *Halomphora* sp*.* |
| 110 ± 15 | 44 | *Nitzschia* cf., *Halomphora* sp*.* |
| 115 ± 15 | 45 | - |
| 740 ± 110 | 71 | *Nitzschia* sp. |
| 1060 ± 110 | 107 | - |
| 3340 ± 120 | 232 | *Planctonema* cf*.*, *Nitzschia* sp.*, Halomphora* sp |
| 6800 ± 140 | 382 | *Halomphora* sp. |

**Supplemental Material Table 5**: Data for statistics for growth rate and oxygen production separated for the temporal cohorts. SD = standard deviation. See Fig. 3 A and B.

| **Temporal cohort from year**  **(cal yr BP)** | **-68 ± 2** | **-13 ± 5** | **110 ± 15** | **1060 ± 110** | **3340 ± 120** | **6800 ± 140** |
| --- | --- | --- | --- | --- | --- | --- |
| **Growth rate (µ d^-1^)** | | | | | | |
| n | 4 | 4 | 4 | 7 | 3 | 8 |
| mean ± SD | 0.30 ± 0.08 | 0.26 ± 0.07 | 0.27 ± 0.10 | 0.31 ± 0.05 | 0.31 ± 0.06 | 0.31 ± 0.06 |
| **Oxygen production (O_2_ mg^-1^ chl-*a* h^-1^)** | | | | | | |
| n | 3 | / | / | 4 | 3 | 4 |
| mean ± SD | 199.25 ± 89.63 |  |  | 224.41 ± 13.93 | 135.82 ± 24.24 | 168.68 ± 70.67 |

**References:**

Dellwig O, Schnetger B, Meyer D, Pollehne F, Häusler K, Arz HW. Impact of the major Baltic inflow in 2014 on manganese cycling in the Gotland Deep (Baltic Sea). *Front Mar Sci* 2018; **5**: 248.

Godhe A, Härnström K. Linking the planktonic and benthic habitat: Genetic structure of the marine diatom *Skeletonema marinoi*. *Mol Ecol* 2010; **19**: 4478–4490.

Häusler K, Dellwig O, Schnetger B, Feldens P, Leipe T, Moros M, et al. Massive Mn carbonate formation in the Landsort Deep (Baltic Sea): Hydrographic conditions, temporal succession, and Mn budget calculations. *Mar Geol* 2018; **395**: 260–270.

Moros M, Kotilainen AT, Snowball I, Neumann T, Perner K, Meier HEM, et al. Is ‘deep-water formation’ in the Baltic Sea a key to understanding seabed dynamics and ventilation changes over the past 7,000 years? *Quat Int* 2020; **550**: 55–65.

Scott C, Lyons TW. Contrasting molybdenum cycling and isotopic properties in euxinic versus non-euxinic sediments and sedimentary rocks: Refining the paleoproxies. *Chem Geol* 2012; **324**–**325**: 19–27.
